# Diagnostic and prognostic roles of echocardiography and cardiac magnetic resonance

**DOI:** 10.1007/s12350-016-0595-z

**Published:** 2016-07-29

**Authors:** Victoria Delgado, Chiara Bucciarelli-Ducci, Jeroen J. Bax

**Affiliations:** 1 Department of Cardiology, Heart & Lung Center, Leiden University Medical Center, Albinusdreef 2, 2300 RC Leiden, The Netherlands; 2 Bristol Heart Institute, University Hospitals Bristol NHS Foundation Trust, Bristol, United Kingdom; 3 Clinical Research and Imaging Centre (CRIC) Bristol, University of Bristol, Bristol, United Kingdom

**Keywords:** Echocardiography, magnetic resonance imaging, sudden cardiac death

## Abstract

Accurate prediction of sudden cardiac death due to ventricular arrhythmia remains challenging. Left ventricular ejection fraction has shown an association with increased risk of ventricular arrhythmias and is included in the recommendations for implantable cardioverter defibrillator as primary prevention. However, left ventricular ejection fraction may be normal in a large number of patients who are at risk of ventricular arrhythmias. Echocardiography remains the imaging technique of first choice to rule out the presence of structural heart disease and assess left and right ventricular function. Advances in strain echocardiography and cardiac magnetic resonance have provided important insights into the mechanisms of ventricular arrhythmias, and will be summarized in this review.

## Introduction

Accurate prediction of sudden cardiac death (SCD) due to ventricular arrhythmia remains challenging. Predictive models should take into consideration the interaction between vulnerable substrates, defined by the presence of genetic or acquired electrical, functional or structural heart disease, and the multiple transient factors (such as ischemia, catecholamine, or electrolyte dysregulations) that may precipitate the ventricular arrhythmia, making almost impossible to obtain one common predictive model for all patients, including individuals without known heart disease. Of the total number of SCD events, almost 50% occur in subjects without known heart disease.^1^ However, many of those individuals have subclinical coronary artery disease and accordingly, risk-profiling strategies that promote preventive and lifestyle modification therapies that reduce the risk of coronary artery disease have been encouraged.^2^ In patients with known ischemic heart disease and dilated cardiomyopathy, left ventricular ejection fraction (LVEF) has consistently shown an association with increased risk of ventricular arrhythmias and therefore, this variable is included in the recommendations for implantable cardioverter defibrillator (ICD) as primary prevention (class I).^3^ However, one third of these patients does not develop ventricular arrhythmias after ICD implantation.^4^ Finally, in patients with inheritable arrhythmogenic diseases, LVEF may be preserved in a large majority of patients and other variables such as duration of the corrected QT interval (in long QT syndrome), interventricular septum thickness (in hypertrophic cardiomyopathy), or right ventricular aneurysms (in arrhythmogenic dysplasia of the right ventricle) have been included in the predictive models.^3^

Cardiac imaging has developed several indices beyond LVEF that permit the identification of patients at high risk for SCD. Echocardiography remains the imaging technique of first choice to rule out the presence of structural heart disease and assess left and right ventricular functions. Advances in strain imaging have provided important insights into the dispersion of the mechanical activation throughout the left ventricle and the presence of heterogeneous regional function that may increase the risk of ventricular arrhythmias.^5^-^8^ Furthermore, cardiac magnetic resonance (CMR) is currently considered the reference standard for the measurement of the cardiac chamber dimensions and function and provides the unique opportunity of noninvasive myocardial tissue characterisation by identifying the presence and extent of myocardial oedema/inflammation, as well as focal, replacement, and interstitial myocardial fibrosis which can be a substrate for arrhythmia.

This review article provides an overview on current evidence showing the additional role of advanced echocardiography and CMR techniques to select patients for ICD implantation for primary prevention. Several echocardiographic and CMR-derived parameters characterizing the arrhythmogenic substrate and transient factors that may increase arrhythmogenicity in ischemic and nonischemic cardiomyopathies will be reviewed.

### Ischemic Cardiomyopathy

In ischemic cardiomyopathy, the most frequent underlying mechanism of ventricular arrhythmia/fibrillation is reentry. The presence of unexcitable dense scar tissue (core infarct) forms an area of fixed conduction block whereas the surrounding areas with viable myocardium intermingled with fibrous tissue (border or peri-infarct zone) increase the nonuniform anisotropy, favors electrical uncoupling and leads to areas of unidirectional conduction block and slow conduction forming the substrate for reentry.^9^ Ischemia acts as a trigger of reentry by enhancing the electrical heterogeneity of the tissue, prolonging the duration of the action potential, influencing the calcium handling and myocyte membrane properties, reducing the cellular coupling and inducing redistribution of connexines.^9^ Furthermore, sympathetic innervation plays a role in the development of ventricular arrhythmias, and it has been shown that patients with ischemic heart disease and large mismatch between the denervated ventricular myocardium and viable myocardium have high risk of ventricular arrhythmic events.^9^ Although, LVEF remains the main parameter to consider ischemic heart failure patients for ICD in primary prevention, it does not reflect the complexity of the arrhythmogenic substrate and transient factors that may trigger the arrhythmias. While late gadolinium contrast-enhanced (LGE) CMR provides high spatial resolution data to characterize the infarct tissue and the components of the arrhythmogenic substrate (infarct core and border zone), several advanced echocardiographic parameters that characterize the functional properties of that substrate and have been associated with the increased risk of ventricular arrhythmias, have been proposed (Table 1).

**Table 1:** Cardiac magnetic resonance and echocardiographic parameters (beyond LVEF) associated with ventricular arrhythmias in ischemic heart failure patients

Imaging technique	Parameter	Evidence
LGE CMR	Infarct size	Increasing number of LV segments with transmural myocardial infarction was associated with increased risk of having appropriate ICD shock (HR 1.48, 95% CI 1.18–1.84, *P* = 0.001)^11^
	Border zone	Each 10-g increase in peri-infarct zone was independently associated with the occurrence of ventricular arrhythmias (HR 1.49, 95% CI 1.01–2.20; *P* = 0.04)^13^
	Conduction channels	Identifiable conduction channels were more frequent among patients with ventricular arrhythmias^17^
Balanced steady-state free precession CMR	Iron deposits-hemorrhage	The presence of hypointense areas within the infarct core, indicating iron deposits or hemorrhage has incremental value to LVEF to predict the occurrence of ventricular arrhythmias^18^
Vasodilator stress CMR	Inducible ischemia	The presence of reversible perfusion defects has prognostic value complementary to LGE for prediction of cardiac death^19^
Echocardiography—Speckle tracking	LV GLS	Reduced magnitude of LV GLS was associated with 1.24-fold increased risk of ventricular arrhythmias (95% CI 1.10 to 1.40; *P* = 0.0004) in 988 patients with acute STEMI^5^
	LV Longitudinal strain—border zone	Each 1% deterioration in longitudinal strain of the LV segments of the border zone was independently associated ventricular arrhythmias (HR 1.22; 95% CI 1.09–1.36; *P* < 0.001) in 424 patients with chronic IHD^8^
	LV mechanical dispersion	Each 10 ms increase in LV mechanical dispersion has been associated with increased risk of arrhythmias in:
		569 patients with acute STEMI/non-STEMI (HR 1.7)^6^
		988 patients with acute STEMI (HR 1.15)^5^
		206 patients with chronic IHD (HR 1.12)^7^
Dobutamine stress echocardiography	Inducible ischemia	The presence of inducible ischemia was associated with ventricular arrhythmias (HR 2.1, 95% CI 1.2–3.5; *P* < 0.001) in 90 patients with chronic IHD^22^

*CI* confidence interval, *GLS* global longitudinal strain, *HR* hazard ratio, *IHD* ischemic heart disease, *LV* left ventricular, *STEMI* ST-segment elevation acute myocardial infarction

**Figure 1:**
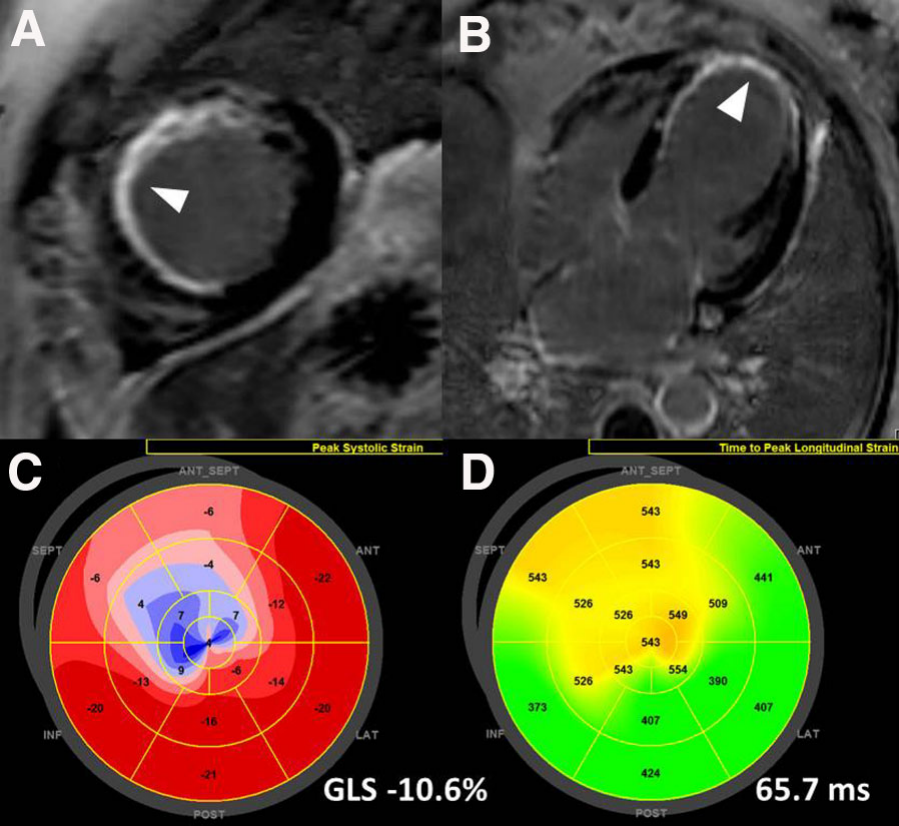
Cardiac magnetic resonance and echocardiographic speckle tracking analysis for risk stratification of patients with ischemic heart disease. Panels **A** and **B** show transmural myocardial scar in the apical septal and anteroseptal segments (*arrows*) and subendocardial scar in the mid-inferoseptal segment. On 2-dimensional speckle tracking echocardiography, the magnitude of global longitudinal strain is −10.6% (panel **C**). The LV apical segments show positive values and are color coded in blue indicating lengthening (correlating with the area of transmural scar). Panel D shows significant mechanical dispersion (65.7 ms) based on the standard deviation of time to peak longitudinal strain of 17 segments. The most delayed areas coincide with the areas with scar and impaired longitudinal strain

In patients with myocardial infarction, gadolinium-based contrast agents accumulate in the increased extracellular space with a subendocardial or transmural distribution within the left ventricular wall reflecting the ischemic-necrotic wave-front phenomenon during myocardial infarction. On T1-weighted CMR acquisitions, myocardial scarring appears hyperintense (white) in contrast to the normal viable myocardium (Figure 1). Detection and quantification of myocardial fibrosis with LGE CMR has been associated with the occurrence of ventricular arrhythmias in patients with ischemic heart disease.^10^-^15^ Scott et al demonstrated in 64 patients with known coronary artery disease who underwent LGE CMR prior to ICD implantation that an increasing number of LV segments with transmural myocardial infarction was associated with increased risk of having appropriate ICD shock during follow-up (HR 1.48, 95% CI 1.18-1.84, *P* = 0.001), whereas LVEF was not.^15^ Furthermore, based on different thresholds of signal intensity compared to normal myocardium (lowest signal) or the infarct core (highest signal), the tissue heterogeneity of the scarred myocardium can be assessed with LGE CMR. The border or peri-infarct zone shows characteristically lower signal intensity than the infarct core but higher than the normal myocardium. This tissue heterogeneity has been associated with the occurrence of ventricular arrhythmias.^13^,^14^ In 91 ischemic heart failure patients receiving an ICD, each 10-g increase in peri-infarct zone was independently associated with the occurrence of ventricular arrhythmias (HR 1.49, 95% CI 1.01-2.20; *P* = 0.04) whereas LVEF and total infarct size were not associated.^13^ In addition, the analysis of the peri-infarct zone with LGE CMR permits the identification of conduction channels (critical isthmus of most ventricular arrhythmias), having important implications for ablation of ventricular tachycardia.^16^,^17^ These channels consist of bundles of viable myocardium surrounded by compact scar tissue that connect with normal myocardium by at least one side of the of the scar and have characteristically a lower signal intensity than the infarct core on LGE CMR images. These structures have been more frequently identified in patients with ischemic cardiomyopathy and ventricular arrhythmias as compared with patients without arrhythmias (88% vs 33%, *P* < 0.001).^17^ The 3-dimensional reconstruction of the myocardial scar can be merged with electroanatomical mapping and facilitate the ablation procedures by noninvasively visualizing the critical isthmus.^16^

The electrophysiological properties of the myocardial scar may change over time and may be influenced by changes in the composition of the extracellular matrix or the presence of ischemia.^18^,^19^ For example, the presence of iron deposition in myocardial infarction has been associated with prolonged inflammation, long-corrected QT interval, isolated late potentials, and LV remodeling.^20^ Iron deposits are visualized on balanced steady-state free precession CMR sequences as hypointense cores and have been shown to provide incremental value to LVEF to predict ventricular arrhythmias (area under the curve 0.68 for LVEF alone, 0.87 for LVEF + hypointense cores).^18^ Furthermore, vasodilator stress CMR perfusion demonstrated the complementary prognostic value of reversible perfusion defects (ischemia) to the presence of LGE.^19^ In 254 patients with suspected or known coronary artery disease (22% with prior myocardial infarction), the presence of reversible perfussion defects was associated with a 3-fold increased risk of cardiac death after adjusting for presence of LGE, age, and gender.^19^

Advanced echocardiography can also assess the tissue heterogeneity of myocardial infarction focusing on the mechanical and electrical properties. Echocardiographic speckle tracking analysis informs about the deformational capacity of the LV myocardium, representing the contractile status of the myocardium. The 3-dimensional disposition of the myocardial fibers changing continuously from a right-handed helix in the subendocardium to a left-handed helix in the subepicardium determines the specific deformation of the left ventricle: shortening-lengthening in the longitudinal and circumferential directions and thickening-thinning in the radial direction.^21^ Myocardial infarction will alter this disposition by the deposition of collagen fibers, scar formation and remodeling increasing the functional heterogeneity of the myocardial tissue of the core infarct, the peri-infarct and the remote zone. The magnitude of global and regional LV longitudinal strain assessed with speckle tracking echocardiography has been associated with the risk of developing ventricular arrhythmias.^5^,^8^ In 988 patients after acute myocardial infarction who were followed up for a median of 28 months, the occurrence of the composite endpoint (SCD, ventricular arrhythmias, or appropriate ICD shocks) was documented in 34 (3.4%) patients.^5^ In this population, Ersboll et al showed that LV global longitudinal strain (GLS) was independently associated with the occurrence of the composite endpoint [hazard ratio (HR): 1.24; 95% confidence interval (CI): 1.10 to 1.40; *P* = 0.0004] (Figure 1). More specifically, regional LV longitudinal strain may better to characterize the function of the infarct core, border zone, and remote zone. Using regional LV longitudinal strain, Ng et al evaluated the prognostic value of the longitudinal strain of the border zone in 424 patients with ischemic heart disease recipients of an ICD.^8^ The infarct core zone was defined by LV segments with a value of regional longitudinal strain of >−5%, and the border zone was formed by all the surrounding segments immediately adjacent to the infarct segments. During a median follow-up of 24 months, 95 (22%) patients had appropriate ICD shocks. When the population was dichotomized according to the medial value of longitudinal strain of the border zone (≤−9.9% versus >−9.9%), patients with values ≤−9.9% (more preserved function) showed less frequently appropriate ICD shocks compared with their counterparts (8%, 11%, and 14% at 1, 2, and 3 years follow-up compared with a respective 15%, 21%, and 25%, respectively; log-rank *P* = 0.008).^8^ On multivariate analysis, each 1% deterioration in longitudinal strain of the LV segments of the border zone was independently associated with 1.22 increased HR of having ventricular arrhythmias (95% CI 1.09-1.36; *P* < 0.001).

In addition, the dispersion of the LV mechanical activation measured with speckle tracking echocardiography as the standard deviation of the time to peak longitudinal strain of 16 LV segments has been proposed as a surrogate to characterize the tissue heterogeneity that may predispose to ventricular arrhythmias (Figure 1).^6^ In a prospective multi-center study including 569 patients who survived >40 days after an acute myocardial infarction (47% with ST-segment elevation myocardial infarction), the prognostic value of LV mechanical dispersion was assessed.^6^ During a follow-up of 30 months, 15 (3%) patients presented with ventricular arrhythmias. Each 10-ms increase in LV mechanical dispersion was associated with increased risk of ventricular arrhythmias at follow-up (HR 1.7, 95% CI 1.2-2.5; *P* < 0.01), whereas LV GLS was not significantly associated. In the study by Ersboll et al above mentioned, LV mechanical dispersion was also associated with increased risk of ventricular arrhythmias (each 10 ms increase: HR 1.15, 95% CI 1.01-1.31; *P* = 0.032).^5^ It has been suggested that LV mechanical dispersion may become more relevant than LV GLS in populations with a history of myocardial infarction.^7^ Indeed, LV mechanical dispersion may be a consequence of scar tissue formation and collagen deposition and may promote itself ongoing ventricular remodeling and fibrosis, particularly in the infarct and border zones. In 206 patients with prior myocardial infarction (median myocardial infarction age, 6.2 years), increasing LV mechanical dispersion was associated with increased risk of ventricular arrhythmias independently of LV GLS (HR 1.12, 95% CI 1.06-1.18; *P* < 0.001).^7^

Furthermore, in ischemic heart failure patients, assessment of myocardial ischemia and viability with dobutamine stress echocardiography is of importance and has been associated with the occurrence of ventricular arrhythmias. In 90 patients with ischemic heart disease and treated with an ICD for primary or secondary prevention, the presence of inducible ischemia during dobutamine stress echocardiography was associated with 2-fold increased risk of death or appropriate ICD therapy at follow-up (95% CI, 1.2-3.5; *P* < 0.001).^22^ Revascularization of ischemic myocardium has demonstrated to reduce the risk of arrhythmic events.^23^

### Nonischemic Cardiomyopathies

Reduction of LVEF in the absence of significant coronary artery disease, valvular heart disease, hypertension, or congenital heart disease, defines nonischemic cardiomyopathy, and can be caused by primary disorders of the myocardium or secondary to systemic diseases that cause myocardial damage.^24^ The most frequent form is dilated cardiomyopathy, with a yearly incidence of 0.57 cases/100,000 per year among children and 7 cases/100 000 per year among adults.^24^ Familial dilated cardiomyopathy may be observed in 20-48% and frequently has an autosomal dominant inheritance. Myocarditis, toxicity-related myocardial damage, metabolic disturbance storage diseases, and infiltrative diseases are other causes of nonischemic cardiomyopathy. Patients with nonischemic cardiomyopathy have an increased risk of ventricular arrhythmias and SCD, and the efficacy of ICD to improve long-term outcome in primary prevention has been shown in several randomized trials.^25^,^26^ Evaluation of arrhythmogenic substrate with CMR and echocardiography may refine risk stratification in patients with nonischemic cardiomyopathy. Replacement fibrosis assessed with LGE CMR and mechanical and electrical tissue heterogeneity using speckle tracking echocardiography have been associated with increased risk of ventricular arrhythmias and SCD (Table 2).^27^-^42^

**Table 2:** Assessment of arrhythmogenic substrate with CMR and advanced echocardiography in nonischemic cardiomyopathies

Imaging technique	Study	No.	Cardiomyopathy	Parameter	Evidence
LGE CMR	Wu et al.^27^	65	DCM	Presence of LGE	Patients with LGE presented more frequently with cardiac death or appropriate ICD therapy (22% vs 8%, *P* = 0.03) Presence of LGE was independently associated with heart failure hospitalization, cardiac death, or appropriate ICD therapy (HR 8.2, 95% CI 2.2-30.9; *P* = 0.002)
	Iles et al.^28^	61	NA	Presence of LGE	Patients with LGE showed significantly higher rates of appropriate ICD therapies compared with patients without LGE (29% vs 0%, *P* < 0.001)
	Lehrke et al.^29^	184	DCM	Presence of LGE Extent of LGE	Presence of LGE was associated with 3.4-fold increased risk of combined end point (cardiac death, appropriate ICD therapy, and heart failure hospitalization) (95% CI 1.26–9, *P* = 0.015) Patients with LGE extending ≥4.4% of the LV mass showed an increased rate in the combined end point
	Gao et al.^30^	65	Myocarditis (*n* = 8) Sarcoidosis (*n* = 6) Chemotherapy (*n* = 3) ARVC (*n* = 1) NA (*n* = 47)	Extent of LGE	Patients with scar mass above the median value (20.8 g) showed higher cumulative risk of appropriate ICD therapy, survived cardiac arrest or SCD than their counterparts (HR 1.8, 95% CI 0.4–7.6; *P* = 0.4)
	Muller et al.^31^	185	DCM (*n* = 102) Myocarditis (*n* = 65) HCM (*n* = 15) Storage disease (*n* = 3)	Presence of LGE	Patients with LGE showed higher cumulative 3-year event rates (composite end point including appropriate ICD and sustained ventricular arrhythmias) than their counterparts (67% vs 27%; *P* = 0.021). However, presence of LGE was not independently associated with outcome (HR 1.1, 95% CI 0.6–2.1; *P* = 0.67)
	Gulati et al.^32^	472	DCM	Presence of LGE Extent of LGE	Patients with mid-wall LGE were 5 times more likely to present with SCD or aborted SCD compared with patients without (29.6% vs 7%). Each 1% increment in LGE extent was independently associated with arrhythmic outcome (HR 1.10, 95% CI 1.05–1.16; *P* < 0.001)
	Neilan et al.^33^	162	NA (infiltrative cardiomyopathy excluded)	Presence of LGE Extent of LGE	Presence of LGE (HR 14, 95% CI 4.4-45.6; p<0.001) and each 1% increment in LGE extent (HR 1.17, 95% CI 1.12–1.22; *P* < 0.0001) were strongly associated with appropriate ICD therapy or non-heart failure cardiac death
	Masci et al.^34^	228	DCM Chemotherapy (n=7)	Presence of LGE	Patients with LGE showed 8.3-fold higher risk of aborted SCD versus patients without LGE (95% CI 1.66–41.55; *P* = 0.01)
	Grün et al.^35^	222	Myocarditis	Presence of LGE Extent of LGE	LGE was more frequently observed among patients who presented with SCD compared with patients without event (100% vs 43%; *P* < 0.001). Presence of LGE was independently associated with cardiac death (HR 12.8; *P* < 0.01)
	Mello et al.^36^	41	Chagas cardiomyopathy	Presence of LGE Extent of LGE	The presence of ≥2 LV segments with transmural scar was independently associated with ventricular arrhythmias (relative risk 4.1; 95% CI 1.06–15.68; *P* = 0.04)
	Kramer et al.^37^	57	Anderson-Fabry’s disease	Presence of LGE Progression of LGE	Only patients with LGE presented with ventricular arrhythmic events. Annual increase in fibrosis (LGE) was the only independent predictor of ventricular arrhythmias (*P* = 0.038)
	Florian et al.^38^	88	Duchnne and Becker muscular dystrophies	Presence of LGE	Presence of transmural LGE was independently associated with heart failure hospitalizations or ventricular arrhythmias (HR 2.89, 95% CI 1.09–7.68; *P* = 0.033)
	Greulich et al.^39^	155	Sarcoidosis	Presence of LGE	Patients with LGE had 31.6-fold increased risk of presenting with death, aborted SCD, or appropriate ICD therapy (*P* = 0.0014)
	Murtagh et al.^40^	205	Sarcoidosis	Presence of LGE Extent of LGE	The annualized rate of death or ventricular tachycardia was significantly higher among patients with LGE compared with patients without (4.93% vs 0.24%, *P* < 0.05). Each 1% increase in LGE extent resulted in 8% increase in the hazard of death or ventricular tachycardia
Speckle tracking echocardiography	Joyce et al.^41^	100	Sarcoidosis	Global LV longitudinal strain	Global LV longitudinal strain was independently associated with 1.4-fold increased risk of composite end point (including arrhythmias)
	Haugaa et al.^42^	94	DCM	Global LV longitudinal strain Mechanical dispersion	Each 1% worsening in global LV longitudinal strain was independently associated with ventricular arrhythmias, SCD and appropriate ICD therapy (HR 1.26, 95% CI 1.03–1.54; *P* = 0.02) Each 10 ms increment in mechanical dispersion was associated with a 1.20 increased risk for ventricular arrhythmias, SCD, and appropriate ICD therapy (95% CI 1.03–1.4; *P* = 0.02)

*ARVC* arrhythmogenic right ventricular cardiomyopathy, *DCM* dilated cardiomyopathy, *CI* confidence interval, *CMR* cardiac magnetic resonance, *HCM* hypertrophic cardiomyopathy, *HR* hazard ratio, *LGE* late gadolinium enhancement, *LV* left ventricular, *NA* not available, *SCD* sudden cardiac death

**Figure 2:**
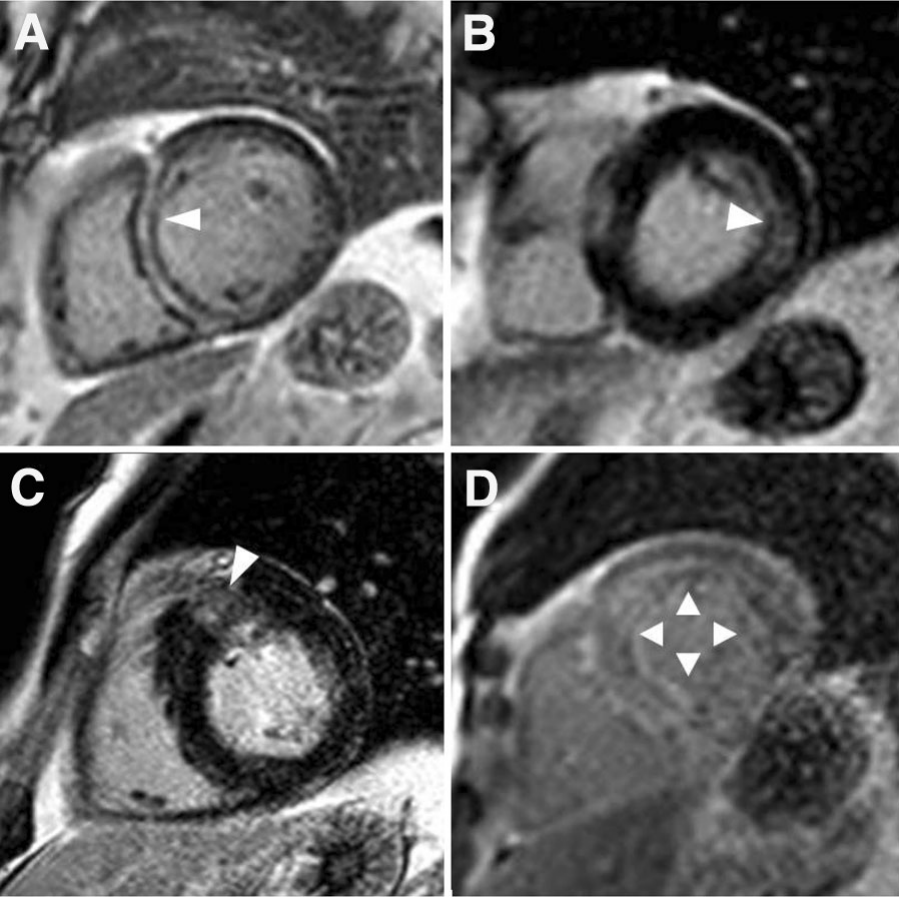
Patterns of late gadolinium contrast enhancement in nonischemic cardiomyopathies. Septal mid-wall late gadolinium enhancement (*arrow*) is typically observed in dilated cardiomyopathy (**A**). Mid-wall late gadolinium enhancement of the basal inferolateral wall (*arrow*) in a patient with cardiac sarcoidosis (**B**). Patchy mid-wall late gadolinium enhancement of the hypertrophic septum at the level of the right ventricular junction (*arrow*) is typical of hypertrophic cardiomyopathy (**C**). In cardiac amyloidosis (**D**), the pattern of late gadolinium enhancement is characterized by circumferential subendocardial distribution (*arrows*)

The association between the presence of LGE (replacement fibrosis) and risk of SCD, aborted SCD, or appropriate ICD therapy for ventricular tachycardia was demonstrated in a recent meta-analysis of 7 studies including 1194 patients with nonischemic cardiomyopathy (odds ratio 5.32; 95% CI 3.45-8.2; *P* < 0.001).^43^ Each type of nonischemic cardiomyopathy may show a distinct spatial distribution of replacement fibrosis on LGE CMR (Figure 2). In dilated cardiomyopathy, 30% of patients may show septal mid-wall fibrosis.^32^ In myocarditis, LGE distribution is typically epicardial, particularly in the inferolateral wall or septum.^35^ Cardiac sarcoidosis and Anderson-Fabry’s disease typically show mid-wall LGE in the basal inferolateral segments, while in Duchenne muscular dystrophy, the distribution of LGE is typically subepicardial affecting the lateral segments.^37^-^39^ Diffuse and patchy distribution of LGE or more typically circumferential subendocardial distribution of LGE can be observed in cardiac amyloidosis.^44^ In contrast to ischemic cardiomyopathy, the different studies evaluating the role of LGE CMR for risk stratification of patients with nonischemic cardiomyopathy have focused mainly on the presence of LGE (Table 2).^27^-^42^ Furthermore, LGE CMR permits characterization of the border zone which may include isthmus sites of ventricular tachycardia in nonischemic cardiomyopathy patients.^45^

Few studies have associated echocardiographic global LV longitudinal strain and mechanical dispersion (as surrogates of myocardial fibrosis and slow conduction) with ventricular arrhythmias and SCD.^41^,^42^ In 100 patients with sarcoidosis who were followed-up during a median of 35 months, Joyce et al demonstrated that global LV longitudinal strain was independently associated with a 1.4-fold increased risk for the combined end point (all-cause mortality, heart failure hospitalization, device implantation, new arrhythmias, and development of cardiac sarcoidosis).^41^ In 94 patients with nonischemic dilated cardiomyopathy, Haugaa and colleagues demonstrated the prognostic value of global LV longitudinal strain and mechanical dispersion.^42^ During a median follow-up, 13% of patients presented with sustained ventricular tachycardia or cardiac arrest. Each 1% worsening in global LV longitudinal strain and each 10-ms increase in mechanical dispersion were both independently associated with a 1.2-fold increased risk of ventricular arrhythmias or cardiac arrest (*P* = 0.002 for both).^42^

### Inheritable Arrhythmogenic Diseases

**Figure 3:**
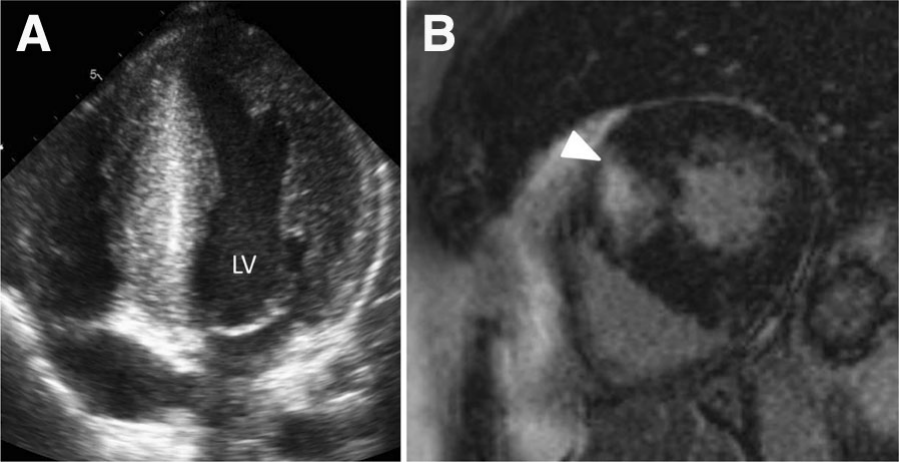
Hypertrophic cardiomyopathy. Panel **A** shows left ventricular (LV) hypertrophy with >15 mm thickness of the septal and lateral walls. Panel **B** shows late gadolinium-enhanced cardiac magnetic resonance of a patient with hypertrophic cardiomyopathy and delayed enhancement in the septum, at the insertion of the right ventricle (*arrow*)

Hypertrophic cardiomyopathy is the most frequent genetic heart disease, usually caused by mutations of genes encoding sarcomeric proteins, transmitted with an autosomal dominant inheritance but with incomplete penetrance and variable expression.^46^,^47^ The diagnosis is based on the presence of abnormally increased LV wall thickness (≥15 mm) by any imaging modality not explained by loading conditions and in the absence of other diseases associated with LV hypertrophy (Figure 3).^48^ The arrhythmogenic substrate is characterized by myocyte disarray and myocardial fibrosis, while microvascular dysfunction, ischemia, and sympathetic innervation disturbances that may influence the arrhythmogenic substrate triggering the occurrence of ventricular arrhythmias. Nonsustained ventricular arrhythmias have been reported in 25% of patients during ambulatory electrocardiographic monitoring^49^ and the prevalence increases with LV wall thickness and the presence of replacement fibrosis on LGE CMR.^50^ The annual incidence of SCD is 0.8%, with the highest prevalence among young patients.^51^,^52^ The 2014 European Society of Cardiology guidelines on the diagnosis and management of patients with hypertrophic cardiomyopathy proposed an algorithm to calculate the risk of SCD.^46^ Maximum LV wall thickness, maximum LV outflow tract gradient (at rest and during Valsalva maneuvres) and left atrial size were included in the algorithm, and can be assessed with echocardiography. Cine CMR can also quantify LV wall thickness and left atrial dimensions; however, quantification of the LV outflow tract obstruction is not routinely assessed. Replacement fibrosis on LGE CMR has been described in 65% of patients (range 33-84%) and is typically distributed following a patchy mid-wall pattern in areas of hypertrophy and at the insertion points of the right ventricle (Figure 3).^53^ In a recent large registry including 1293 patients with hypertrophic cardiomyopathy, each 10% increase in LGE was independently associated with increased risk of SCD events (HR 1.46, 95% CI 1.12-1.92; *P* = 0.002).^54^ The addition of LGE to a SCD event risk model resulted in enhanced integrated discrimination improvement (56.5%) and net reclassification improvement (12.9%). A recent meta-analysis of 6 studies including 3067 patients with hypertrophic cardiomyopathy evaluated with LGE CMR (54% showing LGE) demonstrated that the incidence of SCD events was significantly increased among those patients with LGE compared with patients without (odds ratio 2.52, 95% CI 1.44-4.4; *P* = 0.001).^55^ However, meta-regression analysis showed that the extent of LGE was not significantly associated with SCD events risk (*P* = 0.35) probably due to the inclusion of 5 studies that included patients with a mean LGE extent <10%.^54^,^56^-^59^

**Figure 4:**
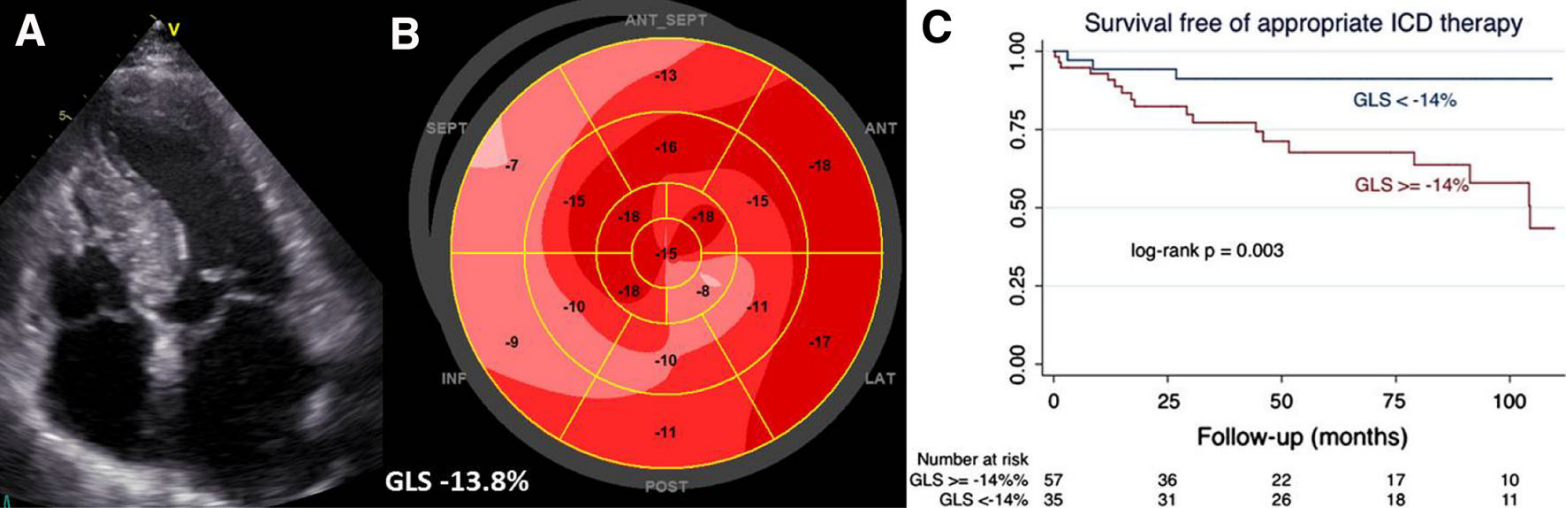
Risk stratification of patients with hypertrophic cardiomyopathy using two-dimensional speckle tracking echocardiography. Example of a patient with hypertrophic obstructive cardiomyopathy with asymmetric septal hypertrophy and systolic anterior motion of the mitral valve (**A**). On echocardiographic speckle tracking analysis, the magnitude of global left ventricular longitudinal strain (GLS) is −13.8% (**B**). The study by Debonnaire et al showed that patients with a left ventricular GLS ≥−14% had higher rates of appropriate implantable cardioverter defibrillator (ICD) therapy compared with patients with more preserved GLS (<−14%) (**C**). Reproduced with permission from Debonnaire et al.^61^

Speckle tracking echocardiographic LV strain analysis has demonstrated to correlate with the amount of replacement fibrosis in hypertrophic cardiomyopathy patients, and therefore it could be hypothesized that the assessment of LV strain may be also associated with increased risk of ventricular arrhythmias (Figure 4).^60^ In 92 hypertrophic cardiomyopathy patients undergoing ICD implantation, Debonnaire et al showed that global LV longitudinal strain measured with speckle tracking echocardiography was independently associated with occurrence of appropriate ICD therapy at follow-up (HR 1.15, 95% CI 1.02-1.3; *P* = 0.03).^61^

**Figure 5:**
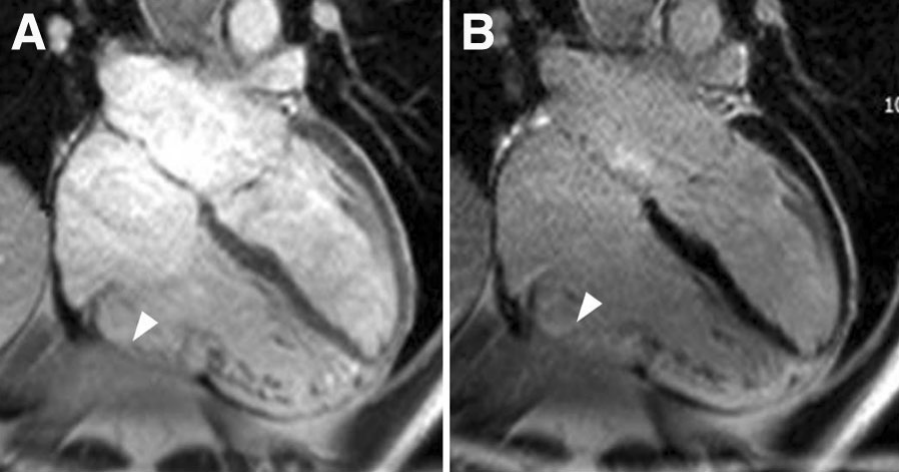
Cardiac magnetic resonance in arrhythmogenic right ventricular cardiomyopathy. Example of a patient who presented with ventricular tachycardia. On cine cardiac magnetic resonance, the 4-chamber view shows a dilated right ventricle, with depressed ejection fraction (35%) and areas of dyskinesia (*arrow*, **A**). On late gadolinium contrast-enhanced cardiac magnetic resonance, the areas with dyskinesia show hyperenhancement (*arrow*, **B**)

Arrhythmogenic right ventricular cardiomyopathy (ARVC) is also an autosomal dominant inheritance disease with variable penetrance and phenotype expression characterized by replacement of ventricular myocardium by fibrous and fatty tissue.^62^ Sustained monomorphic ventricular tachycardia with left bundle branch block morphology is the most frequent arrhythmia and is usually observed at advanced stages of the disease, whereas ventricular fibrillation may occur at any phase of the disease. The imaging criteria that suggest ARVC include right ventricular dilatation and regional right ventricular wall motion abnormalities (aneurysms).^62^ CMR is considered the reference standard for right ventricular volumes quantification and its high spatial resolution permits better identification of right ventricular aneurysms (Figure 5). Two-dimensional echocardiography provides several measurements to accurately estimate the RV dimensions, and with the use of intravenous contrast, the regional wall motion abnormalities can be better visualized. However, it has been shown that echocardiography had lower diagnostic performance compared with CMR.^63^ Although assessment of LGE with CMR in ARVC is challenging due to the thin right ventricular walls and the low specificity (as it can also be observed in other cardiomyopathies that resemble ARVC such as cardiac sarcoidosis), right ventricular LGE can be observed in 88% of patients.^64^ In a recent study including 69 patients with ARVC, the presence of abnormalities on CMR (right ventricular dilatation, wall motion abnormalities, LGE or LV, and biventricular involvement) was associated with development of ventricular arrhythmias; specifically, the presence of right ventricular LGE was only observed in patients presenting with arrhythmic events.^65^ Assessment of global LV longitudinal strain with speckle tracking echocardiography has demonstrated that LV involvement in ARVC patients demonstrated by impaired global LV longitudinal strain was independently associated with the occurrence of ventricular tachycardia, SCD, and appropriate ICD therapies.^66^ If confirmed in larger studies, the results may have important implications, since in many ARVC patients LV involvement occurs at a late stage of the disease, and earlier detection of LV dysfunction may identify the patients who may potentially benefit from an ICD.

Finally, primary electric disorders or channelopathies, such as long QT syndrome, Wolff-Parkinson-White syndrome or Brugada syndrome are characterized by preserved LVEF without structural abnormalities. The prolongation of the action potential and repolarization that occur in these disorders may increase the risk of afterdepolarizations and polymorphic ventricular arrhythmias. Speckle tracking echocardiography has demonstrated to identify mechanical disturbances as a consequence of the electric derangements.^67^ Particularly, in long QT syndrome, LV mechanical dispersion (calculated as the differences in time to regional peak strain measured in the sub- and the midmyocardium) was longer in symptomatic patients compared with asymptomatic carriers (45 ± 13 ms vs 27 ± 12 ms and 46 ± 22 ms vs 26 ± 21 ms, respectively; *P* < 0.001 for both).^67^ There is limited literature on the role of CMR in this patient group. In a cohort of 81 patients with genetically positive Brugada syndrome, CMR demonstrated changes in RV ejection fraction and volumes compared to patients without the mutation.^68^

## New Knowledge Gained

Left ventricular ejection fraction assessed with any imaging technique remains as an important criterion to identify the patients at risk of having ventricular arrhythmias or SCD.^3^ However, a significant proportion of patients with reduced LVEF who receive an ICD for primary prevention may not experience an appropriate therapy. In contrast, patients with relatively preserved LVEF or patients without structural heart disease and normal LVEF may experience life-threatening arrhythmias. Advanced echocardiographic imaging techniques evaluating the active deformation of the myocardium provided the incremental value over LVEF for risk stratification in a variety of patients with cardiac disease. Cardiac magnetic resonance and particularly, the use of LGE have provided further characterization of the arrhythmogenic substrate with important prognostic and therapeutic implications. Current guidelines support the role of these imaging techniques to accurately assess patients at risk of SCD.

## Abbreviations

ARVC: Arrhythmogenic right ventricular cardiomyopathy
CI: Confidence interval
CMR: Cardiac magnetic resonance
GLS: Global longitudinal strain
HR: Hazard ratio
ICD: Implantable cardioverter defibrillator
LGE: Late gadolinium enhancement
LV: Left ventricular
LVEF: Left ventricular ejection fraction
SCD: Sudden cardiac death
